# Anaphylaxis in a 4-year-old male caused by contact with grasses: a case report

**DOI:** 10.1186/s40413-016-0133-0

**Published:** 2017-01-19

**Authors:** Germán Darío Ramón, Victor H. Croce, Iván Chérrez Ojeda

**Affiliations:** 1 Instituto de Alergia e Inmunologia del Sur., Bahia Blanca, Argentina; 2 Hospital Italiano Regional del Sur., Allergy Section, Bahia Blanca, Argentina; 3 Catholic University of Córdoba, Córdoba, Argentina; 4 Universidad de Especialidades Espíritu Santo, School of Medicine, Samborondón, Ecuador; 5 Respiralab, Respiralab Research Group, Guayaquil, Ecuador

**Keywords:** Anaphylaxis, Urticaria, Grasses, Emergency Department, Sensitization

## Abstract

**Background:**

Acute urticaria is the presence of urticaria for <6 weeks, and it is the most common type of urticaria in children. Sometimes, it may be associated with anaphylaxis, a life-threatening condition. Urticaria must be differentiated from anaphylaxis because the latter may require emergency treatment. We describe a child with anaphylaxis exposed to grasses on two occasions.

**Case presentation:**

We described a 4-year-old male child with anaphylaxis exposed to grasses. Patient also suffered mild neurologic/respiratory symptoms but it is unlikely that he had anaphylaxis. Skin-prick tests were positive to *Cynodon dactylis, Phalaris arundinacea and Festuca elatior*. Little is known about the importance of pollens as a cause of urticaria in young children.

**Conclusions:**

The case reported here is particularly interesting because, to the best of our knowledge, anaphylaxis due to pollen exposure in children aged <4 years has not been reported before. We strongly encourage all physicians searching for the cause of acute urticaria (allergists, dermatologists, primary-care physicians) to consider the possibility of pollen allergy, and to screen these patients for pollen sensitization.

## Background

Urticaria is a heterogeneous skin disorder that can be acute or chronic, intermittent or persistent. It may occur alone or in association with other related conditions such as angioedema and, in its most severe form, as part of anaphylaxis [1]. “Acute urticaria” is the presence of urticaria for <6 weeks [2]. It affects ≤20% of the general population, and it is the most common type of urticaria in children [1, 3]. Identification of the cause of pediatric acute urticaria varies widely (20–90% of cases) [4, 5].

Allergic “triggers” are often considered in cases of acute urticaria. However, several other mechanisms by which acute urticaria may occur have been proposed. Infection may be the most common cause in pediatric cases. Hypersensitivity to drugs or food has been reported to be a common potential trigger. In general, urticaria is a self-limiting, innocuous condition but can present with systemic symptoms. It may be associated with anaphylaxis whereby the patient may experience gastrointestinal/respiratory symptoms, or angioedema [6].

Anaphylaxis is a rapid-onset, severe, acute and potentially life-threatening condition. It is characterized by compromise of the airways, breathing/circulatory problems, and is usually (though not always) associated with changes in the skin and mucosa [7]. Key triggers of anaphylaxis include food, drugs, and stinging insects, but in ≤20% of patients the cause is not identified. The relative importance of such triggers varies with age and geography. For presentations to the Emergency Department (ED) of hospitals, drugs and foods are the most common elicitors of anaphylaxis, but differences arise according to age [8].

Urticaria must be differentiated from anaphylaxis because the latter may require emergency treatment, including administration of epinephrine [1]. The Online Latin American Survey on Anaphylaxis (OLASA study) in children aged <5 years revealed the most prevalent trigger agents to be food, drugs and insect stings [9].

Here, we describe a child with anaphylaxis exposed to grasses on two occasions.

## Case presentation

A 4-year-old male was admitted to the ED of our hospital with generalized urticaria. He had been fishing with his father and, 10 min after lying down on grass, he experienced generalized itchy urticaria and angioedema. Thirty minutes later, on examination in the Emergency Department, urticaria was observed over the entire body and angioedema in his hands.

Benadryl and methylprednisolone were administered. Four hours after treatment, symptoms had stopped, and urticaria and angioedema had disappeared completely.

Two weeks later, he visited a farm. Fifteen minutes after lying down on grass he experienced itching sensations in his hands, arms and trunk, along with generalized erythema. He also complained of congestion/itching in his nose, itchy eyes, and crying. His mother stated that he had slight dyspnea with cough and dizziness. She gave him Benadryl and betamethasone via the oral route based on advice proffered in a previous admission to the ED (which improved the most severe symptoms within 1 h). He was admitted to the ED after 2 h with only erythema, and he received antihistamines for an additional 5 days. The patient had no history of atopy.

By anamnesis, an etiology of insect bites, or intake of food or drugs before these two episodes were excluded. Total Immunoglobulin-E was 123 IU/mL, the blood count and complement (CH50, C3, C4, C1 inhibitor) were normal. Parasitology studies were negative. His mother and maternal grandmother reported a history of allergic rhinitis.

**Fig. 1:**
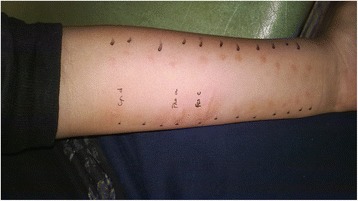
In the SPTs were observed a significantly positive test to *Cynodon dactylis, Phalaris arundinacea and Festuca elatior* (largest diameter (in mm): 8, 12 and 23, respectively), compared with that of the negative control (1 mm)

Skin-prick tests (SPTs; Dermaprick®; Alergo Pharma, Buenos Aires, Argentina) were positive to *Cynodon dactylis, Phalaris arundinacea and Festuca elatior*. Surprisingly these allergens were significantly positive (largest diameter (in mm): 8, 12 and 23, respectively), compared with that of the negative control (1 mm) (Fig. 1). These pollen allergens are used by our research team to ascertain the prevalence of skin sensitivity [10].

SPTs were negative for foods (milk, egg, cocoa, citrus fruits, fish, tomatoes, peanuts, wheat, bananas, strawberries) and airborne allergens (*Dermatophagoides farinae, D. pteronyssinus*, cat dander, dog dander, Alternaria spp., Aspergillus spp., Mucor spp., Cladosporium spp., Penicillium spp., Rhizopus spp., other grasses, weeds, tree pollens).

## Discussion

Hypersensitivity to pollens is very common in adults, and generates a high prevalence of allergic rhinitis and asthma [11], urticaria [12] or atopic dermatitis [13]. However, little is known about the importance of pollens as a cause of urticaria in young children. Most studies have used participants aged 6 or 7 years, and deemed this to be the minimum age for patient cohorts [14–16]. The *rationale* for this strategy is the seasonality of exposure time, prolonging the stage of awareness to the onset of symptoms. Often, etiologic assessment of atopic children excludes pollen antigens in those aged <6 years. Some studies on pollen-based anaphylaxis in children [17] have been carried out.

Anaphylaxis is a severe, potentially fatal, systemic allergic reaction that occurs suddenly after contact with an allergy-causing substance. Our patient have criteria of anaphylaxis in accordance with the major Allergy Societies because had acute onset of symptoms (30 min) with involvement of the skin, respiratory and neurologic compromise [18]. Epinephrine is the treatment of choice for anaphylaxis [19], but in Latin-American it was used in fewer than 25% of anaphylactic reactions [20].

It is known that hospital physicians were not knowledgeable regarding current recommendations for anaphylaxis and this could be the reason why our patient didn’t receive epinephrine for his systemic reactions [21]. Because most of these reactions were treated in EDs, dissemination of anaphylaxis guidelines in this group of physicians should be encouraged [20].

Little is known about pollen sensitization and its potential role in causing urticaria in children. It is known that several types of fruits, vegetables and nuts can cross-react with pollens and cause oral allergy syndrome [22], but our patient did not ingest any of these types of food. Guidelines or review articles mentioning pollen sensitization as a possible cause of urticaria have been reported [2, 5].

An observational cross-sectional study of 280 patients aged 1–10 years showed a positive SPT to pollen in 3% of those aged 1–4 years [23]. One study in asthmatic patients revealed a positive SPT to pollens in 29% of those aged 1–2 years, and 49% in those aged 3–4 years [24].

It had been reported that the prevalence of seasonal allergic rhinitis (SAR) increased markedly after the third birthday, and that the lifetime prevalence of SAR increased significantly from 2.6% at age 2 years to 8.5% at age 5 years, and to 15.3% at age 7 years [25]. In a retrospective study in Latin–American children (6.14 ± 5.1 years) with acute urticaria attending allergy clinics, the SPT for pollens was negative [26].

## Conclusions

Only a few cases of urticaria/angioedema associated with pollen sensitization have been reported. The case reported here is particularly interesting because, to the best of our knowledge, anaphylaxis due to pollen exposure in children aged <4 years has not been reported before.

We strongly encourage all physicians searching for the cause of acute urticaria (allergists, dermatologists, primary-care physicians) to consider the possibility of pollen allergy, and to screen these patients for pollen sensitization. Further studies should be undertaken to determine the prevalence of pollen sensitization in those aged <4 years and the relationship with acute urticaria.

## Abbreviations

ED: Emergency Department
OLASA: Online Latin American Survey on Anaphylaxis Study
SAR: Seasonal allergic rhinitis
SPTs: Skin-prick tests
